# Combining QOF data with the care bundle approach may provide a more meaningful measure of quality in general practice

**DOI:** 10.1186/1472-6963-12-351

**Published:** 2012-10-08

**Authors:** Carl de Wet, John McKay, Paul Bowie

**Affiliations:** 1 Department of Postgraduate General Practice Education, NHS Education for Scotland, 2 Central Quay, Glasgow, Scotland, G3 8BW, United Kingdom

## Abstract

**Background:**

A significant minority of patients do not receive all the evidence-based care recommended for their conditions. Health care quality may be improved by reducing this observed variation. Composite measures offer a different patient-centred perspective on quality and are utilized in acute hospitals via the ‘care bundle’ concept as indicators of the reliability of specific (evidence-based) care delivery tasks and improved outcomes. A care bundle consists of a number of time-specific interventions that should be delivered to every patient every time. We aimed to apply the care bundle concept to selected QOF data to measure the quality of evidence-based care provision.

**Methods:**

Care bundles and components were selected from QOF indicators according to defined criteria. Five clinical conditions were suitable for care bundles: Secondary Prevention of Coronary Heart Disease (CHD), Stroke & Transient Ischaemic Attack (TIA), Chronic Kidney Disease (CKD), Chronic Obstructive Pulmonary Disease (COPD) and Diabetes Mellitus (DM). Each bundle has 3-8 components. A retrospective audit was undertaken in a convenience sample of nine general medical practices in the West of Scotland. Collected data included delivery (or not) of individual bundle components to all patients included on specific disease registers. Practice level and overall compliance with bundles and components were calculated in SPSS and expressed as a percentage.

**Results:**

Nine practices (64.3%) with a combined patient population of 56,948 were able to provide data in the format requested. Overall compliance with developed QOF-based care bundles (composite measures) was as follows: CHD 64.0%, range 35.0-71.9%; Stroke/TIA 74.1%, range 51.6-82.8%; CKD 69.0%, range 64.0-81.4%; and COPD 82.0%, range 47.9-95.8%; and DM 58.4%, range 50.3-65.2%.

**Conclusions:**

In this small study compliance with individual QOF-based care bundle components was high, but overall (‘all or nothing’) compliance was substantially lower. Care bundles may provide a more informed measure of care quality than existing methods. However, the acceptability, feasibility and potential impact on clinical outcomes are unknown.

## Background

Wide variation in the provision of evidence-based care is recognized as a fundamental issue in all health care systems worldwide. The consequences of such variation often impact negatively on patients in terms of health care quality, safety, experiences and increased financial costs (including additional treatments and litigation) associated with sub-optimal clinical practices [1,2].

It is widely accepted that there is a need to minimize unnecessary variation to improve the reliability of best practice care provision and the associated financial costs [2-4]. Practicing evidence-based medicine and implementing clinical care guidelines are promoted to assist clinical decision making and optimal management of patients, but does not necessarily ensure that patients who should receive all appropriate care actually do so [5-8].

Around 50% of hospital patients may receive the full recommended care and treatments which their clinical condition merits. The difference between the highest and lowest performing health care systems suggests that there is ‘an enormous gap’ in evidence-based (or recommended) care provision [1,9]. In primary care settings, evidence of wide variation has also been found between individual health care providers [10]. For example, in general medical practice in the United Kingdom (UK) substantial variation in patient care has been described in anxiolytic, hypnotic, antidepressant and antibiotic prescribing [11-13].

The Quality & Outcomes Framework (QOF) is a pay-for-performance (P4P) scheme that was introduced to UK general practice in April 2004 to help address longstanding variation in the quality of primary care provision [14]. Clinical conditions are suitable for QOF inclusion and therefore financial incentivisation if they are common, associated with significant morbidity (and to a lesser extent mortality), and are diagnostically unambiguous. Indicators should be evidence based, achievable by every primary care team, clearly defined and consistently extractable from different computerized information systems [14].

Since 2009, QOF indicators have been developed through a rigorous National Institute for Clinical Excellence (NICE) led process which includes input from an expert panel and extensive piloting [14]. The Framework consists of a number of incentivized ‘point-in-time’ indicators arranged into four main groups: additional services, patient experience, organizational and clinical sections [15]. Practices ‘earn’ points according to their level of achievement for each indicator, with payment currently starting at a minimum threshold (usually 40%) rising to a maximum (usually 90%).

The average achievement of available QOF points for the period April 2009 to March 2010 was 93.7% in general practices in England and 97.2% in Scotland [16,17]. The implication is that the quality of care delivered by practices to patients with incentivized disease conditions is very high. However, there is some concern that maximum payment thresholds for QOF indicators are actually too low and that the high performances achieved by most practices may give the inaccurate impression that care quality does not necessarily need to be improved further [18,19].

There is growing interest in the use of composite – as well as individual - measures of care delivery as an alternative method of describing the quality of clinical care processes and outcomes [10]. The main benefit of the composite (‘all or nothing’) approach is that it may highlight opportunities for further improvement in care provision even when individual measures already indicate that care quality is high.

The care bundle concept is one such composite measure that is promoted as a systematic method of monitoring and improving the reliability and quality of health care [20-22]. A care bundle is simply a number of health care interventions grouped together and which normally have a synergistic relationship that impacts on clinical outcome [23]. Bundles usually contain three to six components which may include clinical interventions such as care processes, procedures, or diagnostic tests, but are not deemed suitable to act as comprehensive lists of all possible care. Selection of appropriate bundle components is based on best evidence, local considerations and may change with time and experiences [24,25].

Specific care bundles have been implemented in a range of secondary care settings such as paediatric and adult ICU, medical and surgical wards and Accident and Emergency departments in North America and the UK [26,27]. Related clinical outcomes have included significant reductions in health care acquired infections, condition-specific and all-cause mortality, and reduced re-admission rates of elderly patients, length of ICU stay and number of ventilation days [20,28-30]. Although higher compliance rates with bundles are associated with improved outcomes [31], these are difficult to sustain because of a combination of system and human factors which often results in rates below 50% [32-34].

Measuring compliance with bundled interventions on a composite ‘all-or-nothing’ basis may provide the healthcare team with a more accurate indicator of care quality and evidence-based care provision [35]. In essence this means every relevant care component should routinely be delivered (or considered) for every single patient on time and every time. Embracing this rationale may act as a greater prompt to improve patient care than the current method of monitoring data with individual bundle elements, which can give a misleading impression of overall performance.

Evidence of the potential value of composite measures of care quality in general practice – specifically QOF data - is limited [36,37]. Given the benefits of the bundle approach in acute hospital settings, we aimed to develop care bundles based on those QOF disease areas and indicators in general practice that were judged to be most suitable. We further aimed to measure individual and composite compliance with developed care bundles to highlight the extent of any potential care ‘gaps’ which may point to opportunities for further improvement.

## Methods

### Design of care bundles as proxies for composite measures

**Figure 1:**
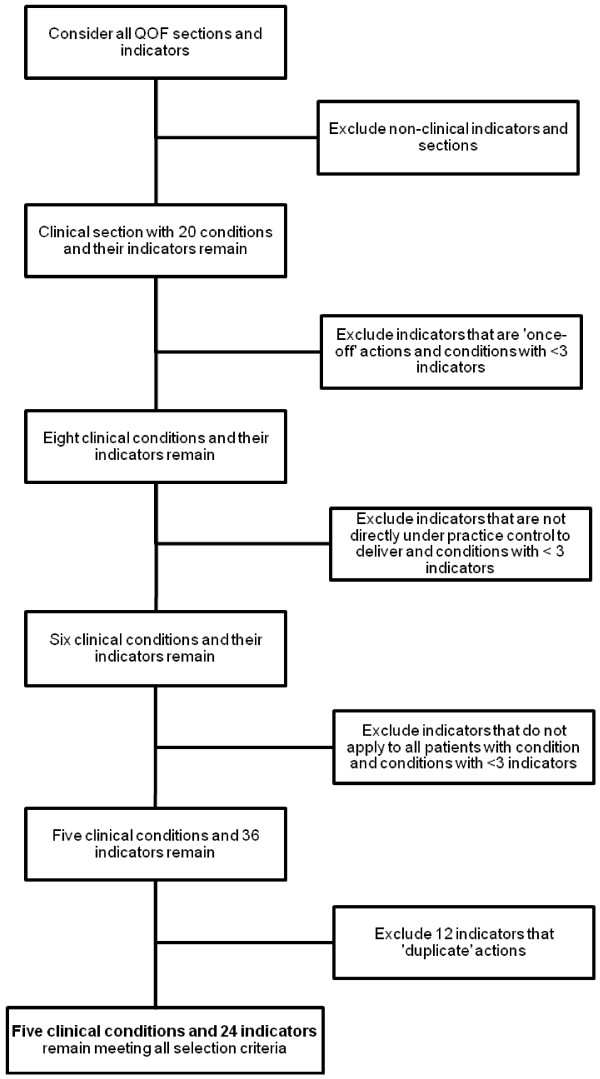
Step-by-step process to select suitable clinical conditions (care bundles) and QOF indicators (bundle components).

The condition-specific care bundles and associated individual components were selected from the ‘Quality and outcomes framework guidance for GMS contract 2009/10’ by the authors [15]. Studying QOF data was a pragmatic choice, as it provided a measure of current evidence-based clinical practices. We agreed selection criteria to help identify potentially suitable clinical conditions and indicators according to previous published guidance [24] and local practical considerations. All QOF sections and indicators were initially considered. The selection criteria (Table 1) were applied in a step-wise manner as illustrated in Figure 1 to exclude unsuitable indicators and conditions.

**Table 1:** Care bundle selection criteria

·	A care bundle should relate to a specific clinical condition
·	A care bundle should have a minimum of three components
·	Care bundle components should describe a specific, measurable action
·	Delivery of every component should be possible for the practice team.
·	Bundle components should be relevant to all patients with that condition.
·	Components must be repeatable, rather than ‘one-off’ actions.
·	Components should not duplicate or be a necessary part of each other

### Measuring care bundle compliance

#### Setting and sample size

We emailed a convenience sample of 14 West of Scotland general practitioners (GPs) who are also senior medical educators based in the west region of NHS Education for Scotland (NES) with details of the proposed study during February 2011. We invited them to discuss the invitation with their practice teams and indicate to us a preparedness to participate.

#### Data collection

On the 31^st^ March 2011 the practice manager in each participating practice identified and downloaded the relevant QOF data requested (using guidance provided) from local information systems to an encrypted USB mobile data device. This device was then transported by the GP educator to the regional office for analysis by the research team. The 31^st^ March is the date on which recorded performance by the practice is formally measured against QOF criteria and associated annual earnings calculated. Practice teams work towards this date all year to record and achieve the maximum patient care as all indicators are automatically ‘reset’ on the following day - 1^st^ April.

Practice managers were asked to indicate the total number of patients registered on their practice list and on each disease register. We decided to code ‘exception reported’ indicators as if they had been met - in other words that the specified care *was* ‘delivered’ to patients. We based this premise on the fact that exception reporting has historically accounted for ≤ 1.6% of all QOF costs [38] and we assumed that the suitability of the care described by an indicator would have been considered (if not delivered) for each patient during the exemption process.

#### Statistical analysis

Data were uploaded to and coded in Microsoft Office Excel 2007. All patient and practice identifiers were removed immediately, with practices being coded to enable feedback to be provided. Descriptive statistics were performed using SPSS v.17.0 to calculate each practice’s compliance as a percentage for the selected QOF indicators (bundle component) and the composite compliance for the selected clinical conditions (bundles). Overall compliance with care bundles (composite measures) and their components were calculated after adding the patients on each disease register of all the participating practices together.

## Results

### Care bundles (composite measures) and indicators

Five QOF clinical conditions fulfilled the pre-specified inclusion criteria: Stroke/TIA, secondary prevention of coronary heart disease (CHD), diabetes mellitus (DM), chronic kidney disease (CKD) and chronic obstructive pulmonary disease (COPD). The 24 retained indicators are shown in Table 2.

**Table 2:** Retained clinical conditions and QOF indicators (care bundles and components)

	**Secondary prevention of Coronary Heart Disease (CHD)**
	The percentage of patients with coronary heart disease:
CHD6	…in whom the last blood pressure reading (measured in the previous 15 months) is 150/90 or less
CHD8	…whose last measured total cholesterol (measured in the previous 15 months) is 5 mmol/l or less
CHD9	…with a record in the previous 15 months that aspirin, an alternative anti-platelet therapy, or an anti-coagulant is being taken (unless a contraindication or side-effects are recorded)
CHD10	…who are currently treated with a beta blocker (unless a contraindication or side-effects are recorded)
CHD11	…a history of myocardial infarction (diagnosed after 1 April 2003) who are currently treated with an ACE inhibitor or Angiotensin II antagonist
CHD12	…who have a record of influenza immunisation in the preceding 1 September to 31 March
	**Chronic kidney disease (CKD)**
	The percentage of patients on the CKD register:
CKD3	…whom the last blood pressure reading, measured in the previous 15 months, is 140/85 or less
CKD5	…with hypertension and proteinuria who are treated with an angiotensin converting enzyme inhibitor (ACE – I) or angiotensin receptor blocker (ARB) (unless a contraindication or side effects are recorded)
CKD6	…whose notes have a record of a urine albumin: creatinine ratio (or protein: creatinine ratio) test in the previous 15 months
	**Diabetes mellitus (DM)**
	The percentage of patients with diabetes:
DM2	…whose notes record BMI in the previous 15 months
DM24	…in whom the last HbA1c is 8 or less (or equivalent test/reference range depending on local laboratory) in the previous 15 months
DM9	…with a record of the presence or absence of peripheral pulses in the previous 15 months
DM10	…with a record of neuropathy testing in the previous 15 months
DM12	…in whom the last blood pressure is 145/85 or less
DM15	…with a diagnosis of proteinuria or micro-albuminuria who are treated with ACE inhibitors (or A2 antagonists)
DM17	…whose last measured total cholesterol within the previous 15 months is 5 mmol/l or less
DM18	…who have had influenza immunisation in the preceding 1 September to 31 March
	**Chronic obstructive pulmonary disease (COPD)**
	The percentage of patients with COPD:
COPD10	…with a record of FeV1 in the previous 15 months
COPD13	…who have had a review, undertaken by a healthcare professional, including an assessment of breathlessness using the MRC dyspnoea score in the preceding 15 months
COPD8	…who have had influenza immunisation in the preceding 1 September to 31 March
	**Stroke and transient ischaemic attack (Stroke/TIA)**
	The percentage of patients with:
STROKE6	…a history of TIA or stroke in whom the last blood pressure reading (measured in the previous 15 months) is 150/90 or less
STROKE8	…TIA or stroke whose last measured total cholesterol (measured in the previous 15 months) is 5 mmol/l or less
STROKE12	… with a stroke shown to be non-haemorrhagic, or a history of TIA, who have a record that an anti-platelet agent (aspirin, clopidogrel, dipyridamole or a combination), m or an anti-coagulant is being taken (unless a contraindication or side-effects are recorded)
STROKE10	…TIA or stroke who have had influenza immunisation in the preceding 1 September to 31 March

### Response rate and demographics

A total of 12/14 practices agreed to participate. Nine practices were able to provide data in the format requested to allow meaningful analysis (64.3%). The combined patient population on the 31^st^ March 2010 was 56,948. The prevalence of the selected clinical conditions in all practices was: CKD 3.4% (range 2.5% to 4.9%), stroke/TIA 2.2% (range 1.7% to 2.7%), diabetes mellitus 4.2% (range 3.6% to 5.5%), CVD 4.6% (range 4.2% to 6.2%) and COPD 2.3% (range 0.8% to 3.8%). Further demographic and disease register details of participating practices are shown in Table 3.

**Table 3:** Individual indicator and composite measures of patient care for selected clinical conditions by general practice for the period 01/04/2010-31/03/2011

**Clinical conditions & Indicators**	***Payment***		**Participating general practices – codes A-I**									**Total**
	Points*	***Threshold***	**A**	**B**	**C**	**D**	**E**	**F**	**G**	**H**	**I**	
**All patients = n**		*Min-Max*	**7613**	**2277**	**2893**	**4567**	**12850**	**10939**	**3507**	**4600**	**7702**	**56948**
**CKD n(%)**			246 (3.2)	111 (4.9)	86 (3.0)	151 (3.3)	321 (2.5)	535 (4.9)	97 (2.8)	172 (3.7)	194 (2.5)	1913 (3.4)
CKD3	*11*	*40-70%*	186 (75.2)	72 (64.9)	64 (74.4)	116 (76.8)	255 (79.4)	421 (78.7)	85 (87.6)	85 (87.6)	143 (73.7)	**1472 (76.9)**
CKD5	*9*	*40-80%*	245 (99.6)	110 (99.1)	86 (100)	151 (100)	316 (98.4)	532 (99.4)	96 (99.0)	96 (99.0)	192 (99.0)	**1890 (98.8)**
CKD6	*6*	*40-80%*	211 (85.8)	111 (100)	85 (98.8)	136 (90.1)	282 (87.9)	469 (87.7)	90 (92.8)	90 (92.8)	171 (88.1)	**1707 (89.2)**
**Overall**			**158 (64.2)**	**71 (64.0)**	**64 (74.4)**	**105 (69.5)**	**225 (70.1)**	**380 (71.0)**	**79 (81.4)**	**79 (81.4)**	**129 (66.5)**	**1320 (69.0)**
**STROKE/TIA n(%)**			173 (2.3)	62 (2.7)	75 (2.6)	100 (2.2)	309 (2.4)	248 (2.3)	67 (1.9)	112 (2.4)	129 (1.7)	1275 (2.2)
STR6	*5*	*40-70%*	165 (95.4)	52 (83.9)	67 (89.3)	90 (90.0)	288 (93.2)	226 (91.1)	56 (83.6)	105 (93.8)	118 (91.5)	**1167 (91.5)**
STR8	*5*	*40-60%*	158 (91.3)	48 (77.4)	60 (80.0)	81 (81.0)	283 (91.6)	213 (85.9)	58 (86.6)	95 (84.8)	110 (85.3)	**1106 (86.7)**
STR12	*4*	*40-90%*	166 (96.0)	60 (96.8)	72 (96.0)	96 (96.0)	305 (98.7)	235 (94.8)	65 (97.0)	108 (96.4)	125 (96.9)	**1232 (96.6)**
STR10	*2*	*40-85%*	151 (87.3)	59 (95.2)	73 (97.3)	96 (96.0)	293 (94.8)	234 (94.4)	61 (91.0)	104 (92.9)	124 (96.1)	**1195 (93.7)**
**Overall**			**129 (74.6)**	**32 (51.6)**	**52 (69.3)**	**66 (66.6)**	**256 (82.8)**	**179 (72.2)**	**43 (64.2)**	**84 (75.0)**	**96 (74.4)**	**945 (74.1)**
**Diabetes Mellitus n(%)**			275 (3.6)	120 (5.3)	159 (5.5)	156 (3.4)	520 (4.0)	458 (4.2)	163 (4.6)	181 (3.9)	366 (4.8)	2398 (4.2)
DM2	*3*	*40-90%*	265 (96.4)	120 (100)	159 (100)	151 (96.8)	506 (97.3)	449 (98.0)	159 (97.5)	175 (96.7)	356 (97.3)	**2340 (97.6)**
DM24	*8*	*40-70%*	250 (90.9)	95 (79.2)	140 (88.1)	133 (85.3)	432 (83.1)	359 (78.4)	136 (83.4)	148 (81.8)	296 (80.9)	**1989 (82.9)**
DM9	*3*	*40-90%*	244 (88.7)	119 (99.2)	147 (92.5)	151 (96.8)	475 (91.3)	421 (91.9)	159 (97.5)	165 (91.2)	353 (96.4)	**2234 (93.2)**
DM10	*3*	*40-90%*	244 (88.7)	119 (99.2)	147 (92.5)	150 (96.2)	475 (91.3)	422 (92.1)	159 (97.5)	166 (91.7)	351 (95.9)	**2233 (93.1)**
DM12	*18*	*40-60%*	244 (88.7)	105 (87.5)	144 (90.6)	118 (75.6)	459 (88.3)	375 (81.9)	133 (81.6)	166 (91.7)	329 (89.9)	**2060 (85.9)**
DM13	*3*	*40-90%*	253 (92.0)	119 (99.2)	154 (96.9)	140 (89.7)	484 (93.1)	426 (93.0)	149 (91.4)	153 (84.5)	345 (94.3)	**2244 (93.6)**
DM22	*3*	*40-90%*	269 (97.8)	120 (100.)	157 (98.7)	151 (96.8)	506 (97.3)	442 (96.5)	160 (98.2)	174 (96.1)	361 (98.6)	**2346 (97.8)**
DM15	*3*	*40-80%*	267 (97.1)	116 (96.7)	156 (98.1)	156 (100)	515 (99.0)	458 (100)	160 (98.2)	180 (99.4)	359 (98.1)	**2363 (98.5)**
DM17	*6*	*40-70%*	253 (92.0)	109 (90.8)	145 (91.2)	147 (94.2)	475 (91.3)	396 (86.5)	139 (85.3)	155 (85.6)	313 (85.5)	**2132 (88.9)**
DM18	*3*	*40-85%*	246 (89.5)	118 (98.3)	153 (96.2)	139 (89.1)	492 (94.6)	419 (91.5)	147 (90.2)	162 (89.5)	351 (95.9)	**2227 (92.9)**
**Overall**			**166 (60.4)**	**75 (62.5)**	**103 ( 64.8)**	**84 (53.8)**	**339 (65.2)**	**239 (52.2)**	**82 (50.3)**	**100 (55.2)**	**213 (58.2)**	**1401 (58.4)**
**Sec prev. of CHD n(%)**			317 (4.2)	128 (5.6)	178 (6.2)	204 (4.5)	547 (4.3)	546 (5.0)	189 (5.4)	203(4.4)	327 (4.2)	2639 (4.6)
CHD6	*17*	*40-70%*	300 (94.6)	118 (92.2)	162 (91.0)	174 (85.3)	524 (95.8)	495 (90.7)	166 (87.8)	187 (92.1)	312 (95.4)	**2438 (92.4)**
CHD8	*17*	*40-70%*	294 (92.7)	103 (80.5)	134 (75.3)	186 (91.2)	510 (93.2)	464 (85.0)	155 (82.0)	162 (79.8)	308 (94.2)	**2316 (87.8)**
CHD9	*7*	*40-90%*	293 (92.4)	120 (93.8)	172 (96.6)	199 (97.5)	541 (98.9)	511 (93.6)	172 (91.0)	195 (96.1)	319 (97.6)	**2522 (95.6)**
CHD10	*7*	*40-60%*	295 (93.1)	91 (71.1)	125 (70.2)	184 (90.2)	504 (92.1)	402 (73.6)	131 (69.3)	149 (73.4)	300 (91.7)	**2181 (82.6)**
CHD11	*7*	*40-80%*	312 (98.4)	128 (100)	178 (100)	202 (99.0)	540 (98.7)	539 (98.7)	187 (98.9)	202 (99.5)	325 (99.4)	**2613 (99.0)**
CHD12	*7*	*40-90%*	293 (92.4)	122 (95.3)	172 (96.6)	193 (94.6)	526 (96.2)	524 (96.0)	178 (94.2)	186 (91.6)	313 (95.7)	**2507 (95.0)**
**Overall**			**228 (71.9)**	**62 (48.4)**	**85 (47.8)**	**118 (57.8)**	**348 (63.6)**	**354 (64.8)**	**86 (45.5)**	**71 (35.0)**	**211 (64.5)**	**1690 (64.0)**
**COPD n(%)**			163 (2.1)	72 (3.2)	92 (3.2)	36 (0.8)	286 (2.2)	298 (2.7)	55 (1.6)	176 (3.8)	142 (1.8)	1320 (2.3)
COPD10	*7*	*40-70%*	152 (93.3)	69 (95.8)	85 (92.4)	32 (88.9)	254 (88.8)	273 (91.6)	52 (94.5)	167 (94.9)	74 (52.1)	**1158 (87.7)**
COPD13	*9*	*50-90%*	155 (95.1)	69 (95.8)	85 (92.4)	34 (94.4)	270 (94.4)	280 (94.0)	54 (98.2)	170 (96.6)	137 (96.5)	**1254 (95.0)**
COPD8	*6*	*40-85%*	150 (92.0)	72 (100)	88 (95.7)	35 (97.2)	270 (94.4)	278 (93.3)	53 (96.4)	166 (94.3)	134 (94.4)	**1246 (94.4)**
**Overall**			**140 (85.9)**	**69 (95.8)**	**83 (90.2)**	**30 (83.3)**	**231 (80.8)**	**254 (85.2)**	**50 (90.9)**	**157 (89.2)**	**68 (47.9)**	**1082 (82.0)**

*The number of points per indicator reflects the allocated number available during the study period.

### Composite measures and individual indicator compliance

The minimum and maximum payment thresholds for the selected indicators ranged from 40-50% and 60-90% respectively. All practices achieved the minimum threshold for all indicators and the vast majority also achieved the maximum threshold for most indicators.

The composite measures of the selected conditions (care bundles) varied for all practices and were as follows: CKD 69.0% (range 64.0-81.4%), stroke/TIA 74.1% (range 51.6% to 82.8%), Diabetes mellitus 58.4% (range 50.3% to 65.2%), CVD 64.0% (range 47.8% to 71.9%) and COPD 82.0 (range 47.9% to 95.8%). Care bundles with more components such as DM and CVD generally had lower compliance rates.

The diabetes mellitus care delivered by all practices as specified by individual indicators ranged from 82.9% (DM24) to 98.5% (DM15), yet overall care bundle compliance was substantially lower at 58.4%. In other words, 58.4% of all eligible patients received all the care specified by the bundle and 41.6% of patients did not receive one or more care components. For CVD care, individual indicator achievements ranged from 82.6% (CVD10) to 99.0% (CVD11) with 64.0% of patients receiving all specified care. The COPD care delivery was the most reliable, with 82.0% of patients receiving all components. However, overall the results suggest that the composite bundle approach highlights a greater level of variation within and between practices in evidence based care delivery and arguably therefore provides a more informed and discriminatory measure of care quality than the current, largely homogenous approach. The variation in the individual indicator and composite measures for each clinical condition and practice can be observed in Table 3.

## Discussion

### Main findings

This small study is the first known attempt to measure composite care delivery for specific clinical conditions in UK primary care using a care bundle approach. We identified five suitable clinical conditions for care bundles: COPD, CKD, DM, TIA/Stroke and CHD. Three to eight components were chosen and adapted from QOF indicators for each bundle. Compliance with individual bundle components was generally high, but substantially lower overall for the ‘all or nothing’ bundles. We would suggest that this ‘gap’ in performance may be a more valid reflection of the variation in expected care delivery at the patient level than current individual measures presently indicate.

### The ‘gap‘ between component and care bundle compliance

There are at least five possible reasons for the observed ‘gap’ between individual component and overall bundle compliance. Firstly, the Framework financially incentivizes an individual indicator approach so this becomes the practice focus. Secondly, some components are easier to deliver than others. Thirdly, care bundle compliance (the composite measure) will always have a value equal to or lower than that of the indicator with the lowest performance - consequently there will always be a gap, although it is unknown whether or when the size of the ‘gap’ becomes of clinical significance. Fourthly, the ‘gap’ will usually increase exponentially with the rise in the number of bundle components. Finally, natural variation is inherent in all health care systems and will be affected by the effectiveness and efficiencies of local processes and team efforts.

Variation in care is introduced ‘naturally’ through different and often complex patient presentations and ‘artificially’ through individual clinician differences and priorities, extenuating circumstances and local systems. ‘Patient characteristics’ and ‘deprivation’ have previously been identified as the two factors which are strongly associated with variation (‘the gap’) in primary health care settings in Scotland [12], England [11,13,39], the Netherlands [40], Australia [41] and the USA [42].

The care ‘gap’ between components and care bundles in our study was observed for all clinical conditions and participating practices. This ‘gap’ (variation) may be at least partially accounted for by hidden patient and deprivation factors. The implication may be that the care bundle method may measure the gap but could not necessarily help to eliminate it.

In UK secondary care settings, reported compliance with a variety of clinical care bundles ranges from 19-52% [32,33]. Low compliance rates have important patient safety implications, as a positive and significant association has been found between compliance rates and clinical outcomes such as mortality [27,28,32]. A similar association has not yet been shown for primary care. Although the participating practices’ compliance with all the selected bundles was ≥ 58% it is not known whether compliance can be compared meaningfully across settings and for different care bundles. It would also be desirable to determine to what degree different compliance levels may impact on clinical outcomes in primary care settings.

### The QOF and care bundles

We adapted care bundles from the QOF, currently the most ambitious, comprehensive and largest P4P scheme and quality measure in international healthcare and a ‘natural experiment in progress’ [43]. Framework research and experience may therefore be applicable to our study findings. For example: the Framework can cause a ‘street lamp effect’, i.e. neglect of health care activity and clinical conditions which do not attract financial incentives; it can create a de-personalizing ‘box ticking culture’; it is vulnerable to data distortion and potential gaming; it is accelerating a transition to nurse-led primary care; there is tension between the different QOF roles as a quality improvement method, regulatory framework or remuneration mechanism; the Framework promotes simplicity over complexity and measurability over meaningfulness. These valid concerns could similarly be raised about the care bundle method, with no clear answers at present [19,43-46].

The impact of QOF on clinical outcomes continues to be debated. There is no evidence of any ‘discernable effect’ on hypertension-related outcomes [47], while new depression indicators failed to improve disease detection or treatment [46]. In addition, associations between QOF measures and all-cause mortality and emergency admissions have been found to be ‘small and inconsistent’ [39]. On the other hand, evidence of improved care quality for patients with specific chronic diseases and a ‘trend towards improvement’ in process and outcome at least partly attributable to QOF have recently been reported [44,48-50]. It is unclear what impact (if any) surrogate measures such as care bundles would have on clinical outcomes, how this could be feasibly measured and what period of time would be required.

‘Time-interval’ indicators have been proposed as an alternative and more reliable measure to ‘point-in-time’ indicators to assess care quality [51]. Performance is evaluated over a specified period rather than at a single point in time. Mabotuwana et al applied this approach to hypertension management and found it feasible but with lower measures for ‘interval indicators’ compared with point-in-time indicators. The differences, similarities and potential benefits of any one method, whether care bundles, time-interval or QQF indicators are unclear.

### Care bundle implementation

The care bundle method may be a useful new care quality measure and help to reduce healthcare variation. It may also help to better differentiate the care quality of practices, given that their current QOF scores are now broadly comparable. To implement the bundle a number of challenges would have to be overcome, for example: resistance from practices who are financially disadvantaged by ‘lower’ measures than the current thresholds; accounting for ‘natural’ variation so that practices are not unfairly penalized; existing information technology systems would have to be redesigned; a full evaluation to identify both impact and unintended consequences (e.g. a proportion of patients may end up receiving ‘no care’) would be necessary. If this new measure is considered desirable, it would have to be promoted and incentivized.

Reducing variation in quality can decrease costs if the care ‘gap’ is large, but costs increase as the gap narrows until there is a net expense [52]. Evaluation of care bundle implementation in some secondary care settings has found them to be cost-effective [53]. However, implementing interventions require initial financial and resource investment. McNeill et al found that only 20/265 (12%) of acute medicine units in the UK had the minimum facilities to comply with the ‘surviving sepsis care bundle’ [34]. However, the applicability of care bundles in acute settings cannot be assumed to translate to QOF-bundled components because care packages over 9-15 month periods.

The financial implications of quality-reporting programmes vary greatly. The QOF ‘probably’ represents value for money [54]. Adapting the Framework’s reporting system to allow composite quality measures would require substantial investment and feasibility and acceptability concerns would have to be addressed. Known incentives that facilitate increased practice participation include: financial payments, staff training and providing technical support [54,55].

Implementing care bundles and increasing compliance with them relies not only on individual health care professionals but even more on the availability of adequate resources, support systems and leadership. Aligning care bundles with larger quality improvement initiatives and providing related training also appears to be an important factor influencing success and impact [28]. These issues need to be considered if the care bundle approach is to be successfully implemented in primary care settings.

### Exception reporting and threshold ceilings

The threshold ‘ceiling’ for maximum financial reward is set at ≤ 90% achievement. Practices could hypothetically ‘stop’ delivering care once they reach these thresholds without financial penalty. Our findings indicate that many indicators with relatively low achievement ceilings were still performed for the vast majority of patients which seems to suggest that thresholds are an unlikely reason for the observed ‘gap’.

Exception reporting has previously been found to be consistent across practices and regions and accounts for a tiny minority of cases [38]. Concerns that practices may be ‘gaming’ results through excessive or inappropriate exception reporting have not been substantiated. However, exception reporting combined with less than 100% maximum threshold targets introduce an incentive ceiling [18].

### Strengths and limitations

Our findings are based on a small, convenience sample of practices. We did not take into account patient list size, geographical distribution (degree of remoteness), socio-economic class and level of deprivation or prevalence of disease. Our findings may still have wider application, as different degrees of remoteness, access and practice size do not significantly contribute to variation in care quality as measured by QOF results [56,57]. A recent review found that inequalities in chronic disease management have largely persisted after introduction of QOF (even though quality of care may have increased) [58]. While deprivation is a major contributing cause to overall care, it has not been shown to affect QOF scores. For example, Hamilton et al found that the impact of QOF on diabetes mellitus care was comparable in affluent and deprived areas [59].

We decided to code exception reported indicators as if the described care had been delivered. The consequence was a ‘smaller’ compliance ‘gap’, but one that may have more acceptability to frontline staff. Exclusion systems have an important role to help militate against the potential impact of socio-economic, patient and other factors out-with the practice team’s control. Simple care processes are mitigated more than complex ones and the additional work required in deprived areas is not always being rewarded [60]. We therefore purposefully selected ‘simple’ care bundle components in preference to ‘complex’ ones where possible. We accept that there are many other potential care bundle topics for general practice. However we chose a pragmatic approach to give an initial indication on the feasibility of the concept as applied to QOF data. Others may have chosen alternative approaches and clinical topics for bundles.

## Conclusions

Based on our small study, we believe that the care bundle approach to QOF data has the potential to provide a more insightful measure of the quality of evidence based care provision than the current approach which focuses on compliance with individual indicators rather than individual patients. However, issues around the feasibility and acceptability of the implementation of this method as part of routine general practices need to be explored.

## Competing interests

The authors declare that there are no competing financial or non-financial interests.

## Authors‘ contributions

CdW led on the study design and data collection, undertook statistical analysis and interpretation, and drafted the initial manuscript. JM contributed to study design, data collection and critical review of the manuscript. PB conceived the study idea, acquired funding and contributed to the study design and the drafting and critical review of the manuscript. All authors read and approved the final manuscript.

## Pre-publication history

The pre-publication history for this paper can be accessed here:

http://www.biomedcentral.com/1472-6963/12/351/prepub
